# Empirical investigation of locally made biodiesel on the automobile properties of diesel engine

**DOI:** 10.1016/j.mex.2018.10.020

**Published:** 2018-10-23

**Authors:** M.E. Emetere, A.D. Adejumo, S.A. Adelekan

**Affiliations:** a Department of Physics, Covenant University Canaan Land, P.M.B 1023, Ota, Nigeria; b Department of Mechanical Engineering, Covenant University Canaan Land, Ota, Nigeria; c Mechanical Engineering and Science, University of Johannesburg, APK, South Africa

**Keywords:** Machine testing and biodiesel quality, Biodiesel, Energy, Palm, Diesel engine

## Abstract

Research have been able to resolve the challenges of biodiesel production from whatever source. The ultimate use of locally made biodiesel for domestic automobile engine is the focus of the study. Biodiesel from palm biomass was made using proven laboratory techniques. The derived biodiesel was mixed with petro-diesel in the proportion of B10 and B20. The TD 200 diesel engine was used to estimate automotive outcomes such as torque, rotational speed, differential pressure, operational temperature, power generated, sound of engine, power generated by engine in horse power, thermal efficiency, normal engine efficiency and brake mean effect pressure. The advantage of the experimental method is to:

• Provide a simple and efficient way of analyzing locally made biodiesel.
• Parametrically investigate the pros and cons of biodiesel product.
• Fully understand the impact of biodiesel on the well-being of diesel engines.
• Chat a new course for biodiesel engine design for maximum utilization.

## Graphical abstract

**Figure:**
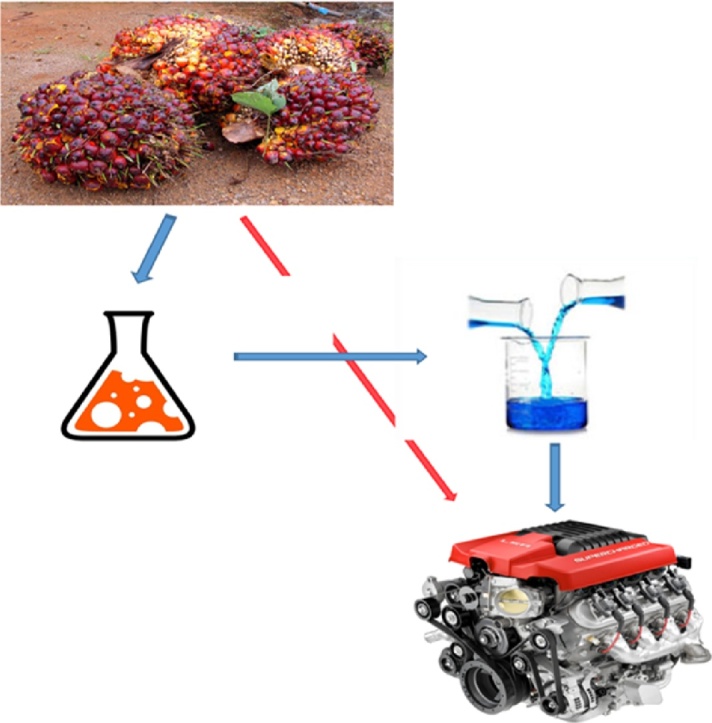

**Specification Table**

**Table:** 

Subject Area	Energy
More specific subject area	Biofuels
Method name	Machine testing and biodiesel quality
Name and reference of original method	Moses Eterigho Emetere, Solomon Jack-Quincy, Akolade Adejumo, Oluwatobi Dauda, Israel Osunlola, Damola Adelekan and Oladipupo Adeyemi (2018). Empirical Analysis of biodiesel effect on the automobile properties of diesel engine: A case study of Olive and Soya biomass, Energy Science & Engineering, 2018: 1-13
Resource availability	ME Emetere, IS Aro, S Jack-Quincy, OD Okonkwo, ME Ojewumi, Omodara J., Jasper N., (2017). Investigating the cyclic breaking of butyl-, methyl-and ethyl-biodiesel from waste vegetable oil using ultraviolet-visible spectrophotometry, Cogent Engineering, 4, 1321084 M.E. Emetere, S. Jack-Quincy, S.I. Aro, O.D. Okonwo, F.T. Owoeye & S.E. Sanni (2017): Validation of biodiesel quality of Monodora myristica and Moringa oleifera using regression and error analysis of UV absorption results, Biofuels, DOI: 10.1080/17597269.2017.1345362

## Method details

### Materials

• reagents (methanol 99%, catalyst (sodium hydroxide (NaOH pellets)).
• thermometer,
• Soxlet apparatus, round bottom flask, flat bottom flask
• 250 and 500 ml conical flask, burette and pipette
• 200 and 500 ml beakers, 200 and 500 ml reagent bottles,
• 250 and 500 ml separation funnels, measuring cylinders
• reflux condenser and rotary evaporator
• funnels and vials
• 500 ml glass jar.
• retort stands, wooden corks and spatula
• plastic containers, potable heat stove and plastic spoon
• heating mantle, hot plate with a magnetic stirrer (magnetic bar)
• digital weighing balance, stop watch and water bath
• density bottles, viscometer tube and viscometer bath
• UV spectrophotometer (2R1R235201)
• TQ data display unit
• TQ TD200 biodiesel test set.
• sound level meter

### Procedure

• The palm biomass is placed in an expeller or a mechanical press.
• The shaft is then transferred to a Soxlet extractor (apparatus) and heated using a heating mantle.
• the oil is boiled to about 110 °C to make sure there is no water present in the oil, then allowed to cool to 40 °C.
• The methoxide was prepared by dissolving 0.4 g of concentrated sodium hydroxide (NaOH) in 20 ml of alkanol (methanol).
• Temperature was regulated to 60 °C.
• The mixture was stirred for 2 h then poured in a separation flask and left for 24 h.
• Biodiesel formed on top, glycerin settled at the bottom.
• Biodiesel was washed about five times to get rid of the glycerin.
• The biodiesel was characterized using the UV Spectrometer at 500 nm, 600 nm, 700 nm, 800 nm, and 900 nm wavelength [1,2].
• The quality and properties of the biodiesel was done using the new methods [3,4].
• The viscosity experiment was also carried using a viscometer and a viscometer bath tube.
• The biodiesel is heated up in the viscometer to a constant temperature of 40 °C. The C value for the viscometer used was 7.870 (mm^2^/sec).
• 10% of the biodiesel product was mixed with 90% pure diesel to give B10.
• 20% of biodiesel was mixed with 80% of pure diesel and this was put in a 1liter bottle after measuring with measuring cylinder.
• The biodiesel mixture (B10 and B20) was fed into the TD 200 test bed engine.
• The speeds were regulated at 2000, 2150, 2169, 2172, 2193, 2200, 2500 revolutions per second.
• The engine torque, rotational speed, differential pressure, operational temperature and power generated measured at the specified engine speed.
• Pure diesel was used as the control of the experiment. The parameter was also derived for the petro-diesel.
• The sound of engine was measured using sound level meter.
• The secondary dataset (power generated by engine in horse power, thermal efficiency, normal engine efficiency and brake mean effect pressure) were derived mathematically from the initial measurements from the TD 200 test bed engine [5,6].
• The dataset was obtained at the TQ data display unit.
• The simulation was carried out using Matlab and CERN Root

### Method validation

500 nm wavelength was selected as the control value during the characterization of the biodiesel produce (using UV spectrophotometer). This idea is to comprehend the changes of the cyclic breaking nature of biodiesel products as postulated in ref [3,4]. The determination of the cyclic breaking in biodiesel depends on the number of clear peaks in the absorption spectra. Sometimes excessive (unclear) peaks indicate the error due to noise in the biodiesel product. The density and viscosity experiment of the biodiesel is presented in Table 1, Table 2 respectively.

**Table 1:** Results obtained from density experiment.

Test fuels	Weight of density bottles	Weight of the test fuels with the density bottle	Actual weight of test fuels	Actual weight /volume of bottle(50 ml) = actual density
Diesel	28.0	71.4	43.4	868
Palm B10	30.5	73.0	42.5	0.85
Palm B20	28.3	71.1	42.8	0.856

**Table 2:** Results obtained from viscosity experiment.

Test fuels	Temperature (^o^C)	Time(sec)	Viscosity (Pa sec)
Palm B20	40	1.35	10.63
Palm B10	40	1.38	10.87
Pure Diesel	40	0.90	7.08

The viscosity of the biodiesel blends was measured as shown in Table 2.

**Fig. 1:**
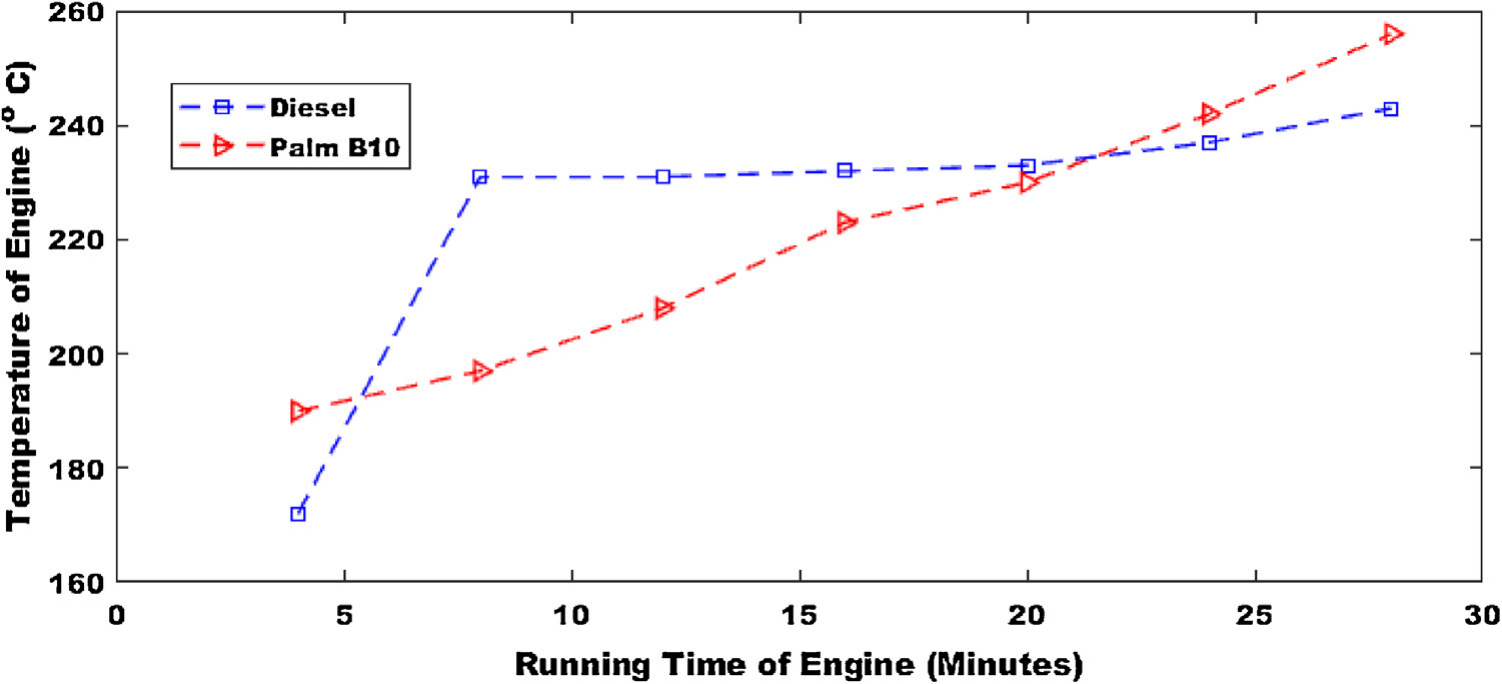
Operational temperature for pure diesel and biodiesel blend of palm B10.

**Fig. 2:**
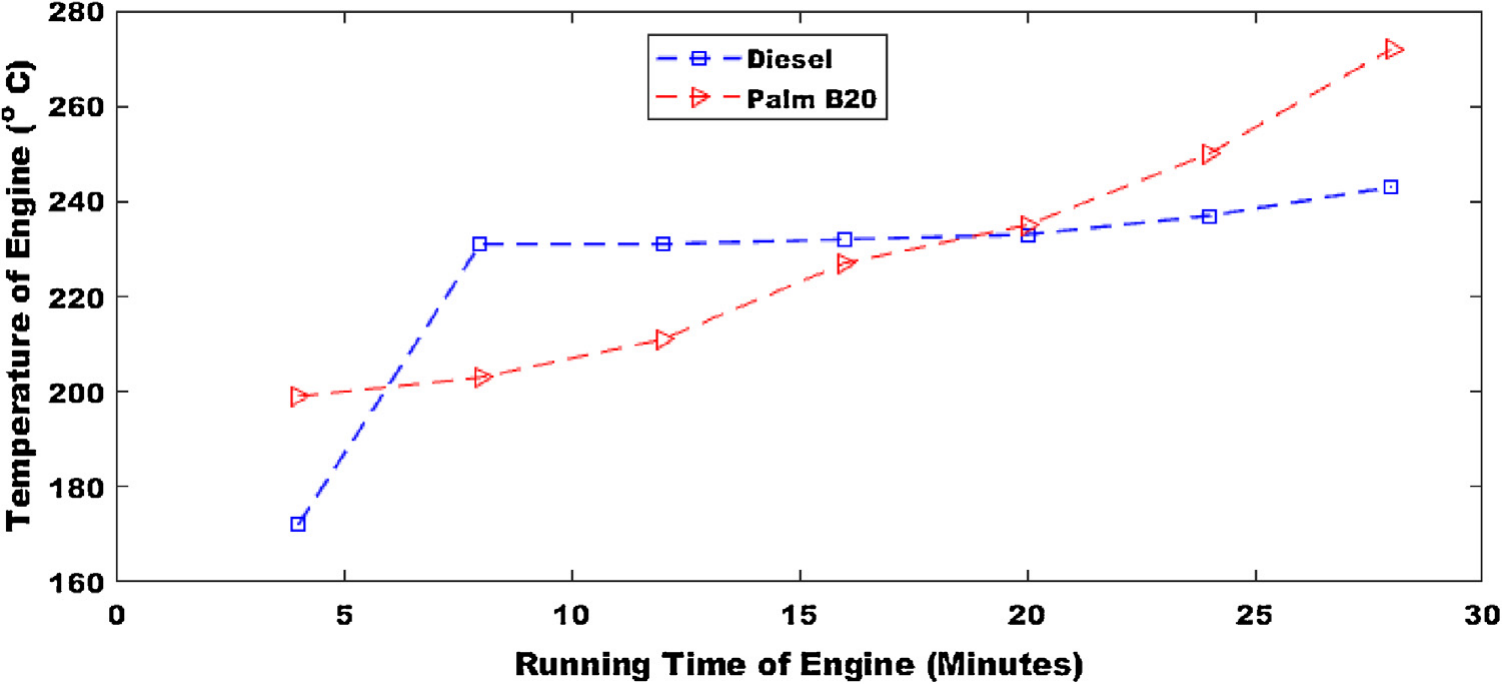
Operational temperature for pure diesel and biodiesel blend of palm B20.

The first parameter that was considered was the operational temperature of the machine as presented in Fig. 1, Fig. 2. The operation temperature of palm B10 had the highest temperature of 246 °C. The pure petro-diesel had a maximum temperature of 258 °C. Hence the temperature of the engine would decrease by 4.65% for palm B10. The operation temperature of palm B20 (shown in Fig. 2) had the highest temperature of 246 °C. The maximum temperature of the pure petro-diesel was 248 °C. This means that the temperature of the engine would decrease by 0.8% for palm B10. This result shows that the combustion rate would be lower for the biodiesel than the petro-diesel.

**Fig. 3:**
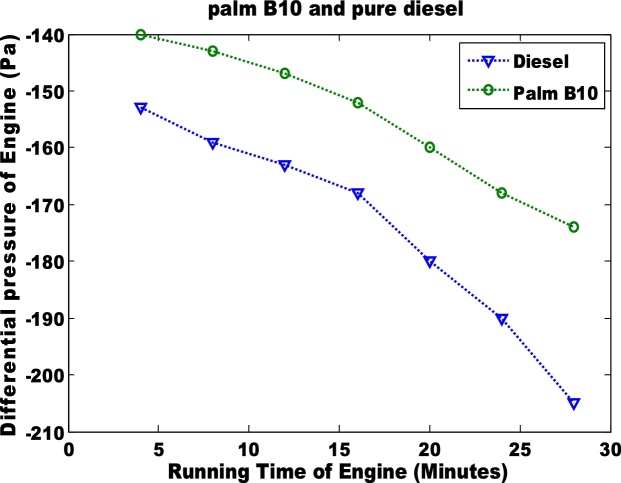
Differential pressure for pure diesel and biodiesel blend of palm B10.

**Fig. 4:**
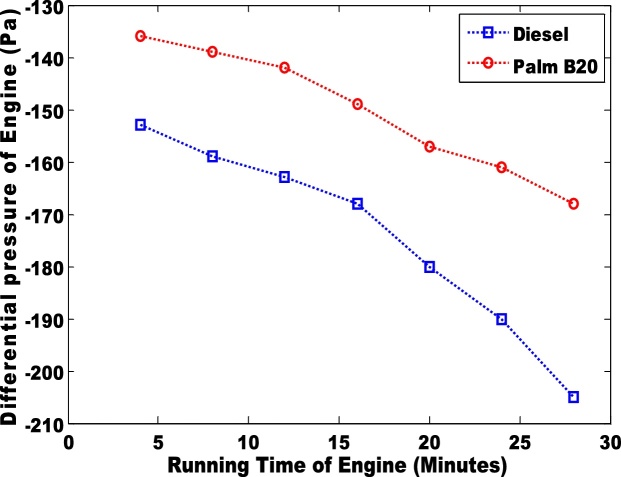
Differential pressure for pure diesel and biodiesel blend of palm B20.

**Fig. 5:**
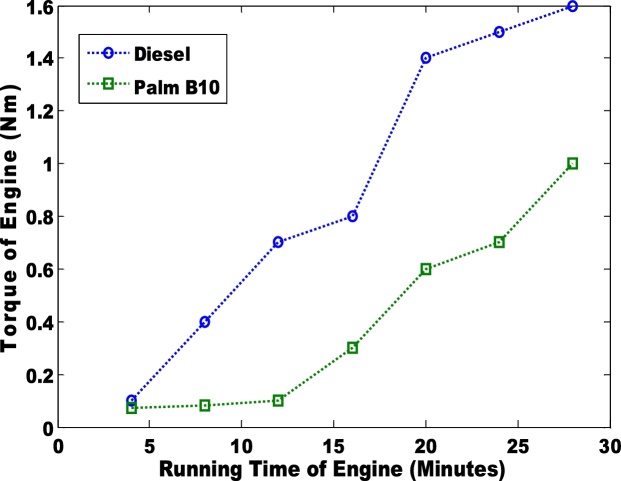
Torque produced by engine for pure diesel and biodiesel blend of palm B10.

**Fig. 6:**
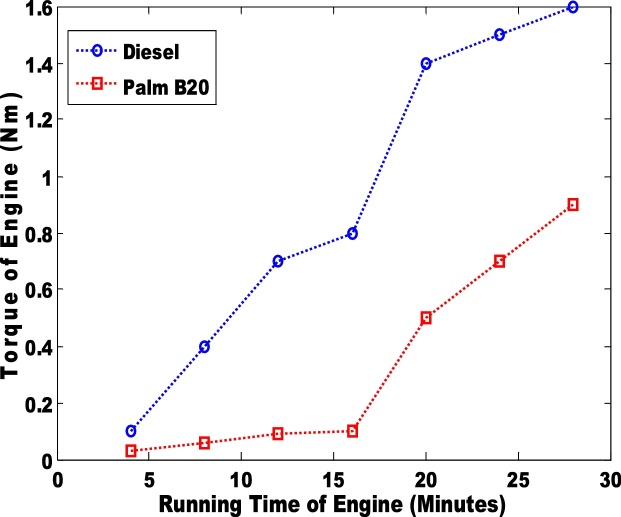
Torque produced by engine for pure diesel and biodiesel blend of palm B20.

The second parameter that was considered was the differential pressure of the engine when biodiesel and petro-diesel products were fed into the engine (Figs. 3 & 4 ). The results were negative. In an engine, the negative differential pressure is caused by depression that is created by the energy transferred from the expansion of gases (during combustion) to the crankshaft via the piston. The seal that is formed between the piston ring and the cylinder wall creates depression as the piston travels downward at a high velocity. Hence, palm B10 had the highest depression. The petro-diesel had a lower depression due to low differential pressure. It can be observed that the differential pressure is higher in B20 than in B10 (Fig. 4). The third parameter that was considered was the torque in the engine (Figs. 5 & 6 ). Petro-diesel had the highest torque. This means that if the engine where to be an automobile engine, the vehicle would accelerate faster than when pure diesel is fed into the engine (shown in Fig. 5, Fig. 6). The wide difference in the torque result may result in losses for the engine. If the engine experience high losses, it may lead to the damage of the piston. This stance is supported by Fontaras et al. [6] who reported that B50 and B100 biodiesel sample caused the wear of some vital parts of an engine because of the presence of iron and copper content present in the biodiesel.

Hence, the group of researcher postulate that the cyclic breaking causes either power or torque losses in engine. The palm B20 biodiesel had lower torque (0.8Nm) than the palm B10 biodiesel (1.01 Nm).

**Fig. 7:**
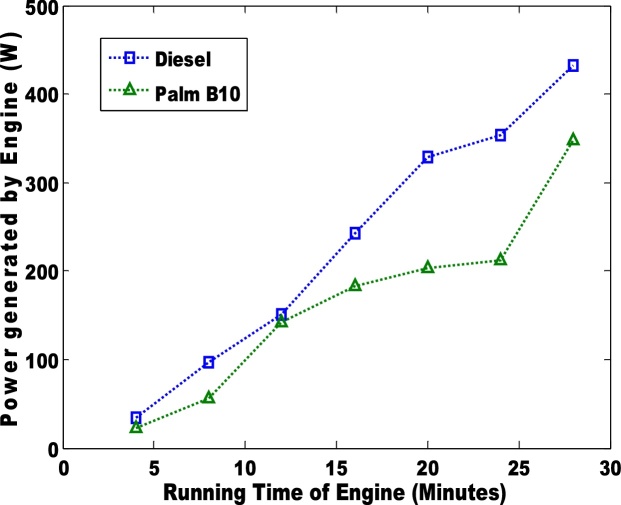
Power generated in the engine for pure diesel and biodiesel blend of palm B10.

**Fig. 8:**
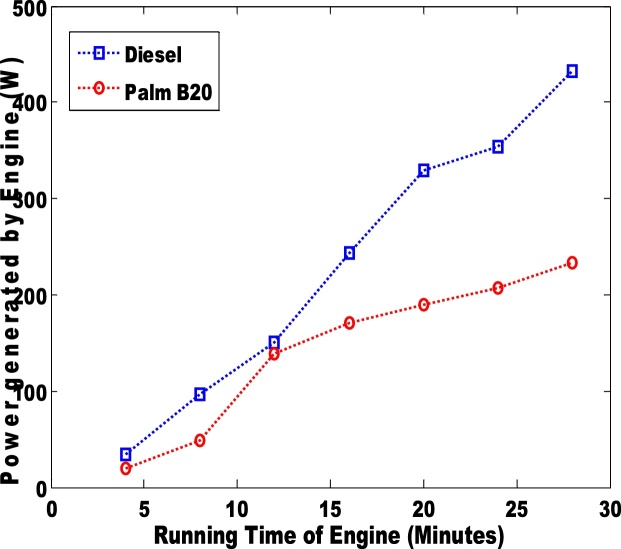
Power generated in the engine for pure diesel and biodiesel blend of palm B20.

**Fig. 9:**
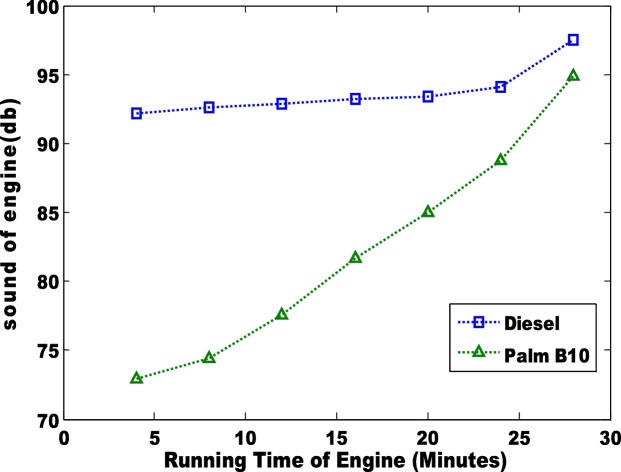
sound of engine using pure diesel and biodiesel blend of palm B10.

**Fig. 10:**
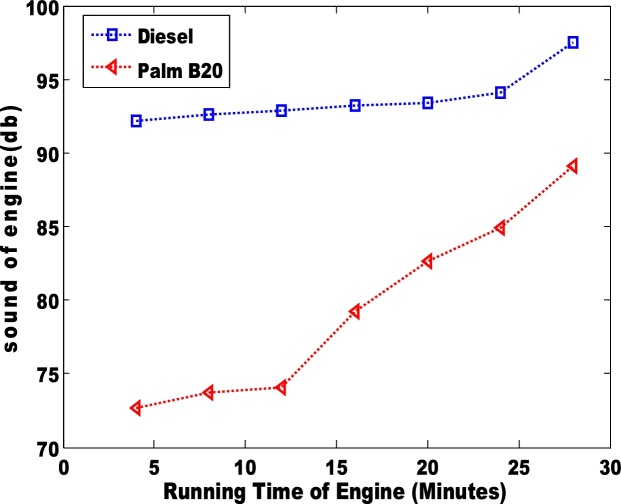
sound of engine using pure diesel and biodiesel blend of palm B10.

The power generated via the use of diesel and biodiesel blend is presented in Fig. 7, Fig. 8. The palm B10 generated higher power than the B20. Unlike the power generated by the engine, the power loss to the passive area is more distinct. The petro-diesel had the highest loss of 10. 3% which is slightly higher than the observation (6.7%) of Murillo et al. [7]. The sound of the engines when the pure diesel and biodiesel blends (B10 and B20) were used is shown in Fig. 9, Fig. 10. The low sound of the engine show that the engine has been stretched out of its limit. However, the B10 biodiesel showed higher magnitude of sound energy than the B20. There are factors that affect the sound of a diesel engine e.g. bore size, stroke length, and crankshaft angles etc. This research considers the stress of the piston as it related to the input fuel. Hence, total work done by the engine is significantly reduced when the engine is working on either blends of biodiesel. Therefore, the general performances of the biodiesel samples (B10 and B20) in the TD 200 test-bed engine may not be adequate based on the sound of the engine.

## Additional information

**Fig. 11:**
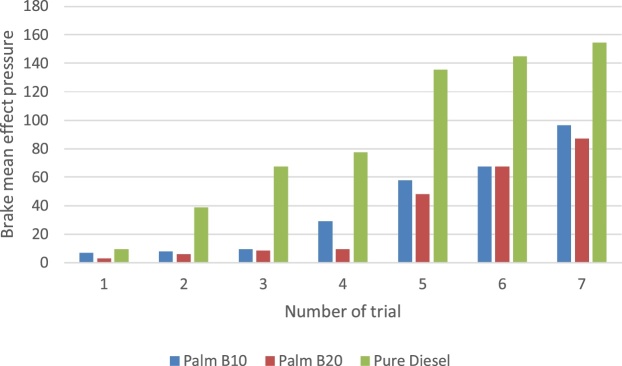
The break mean effect pressure of fuel samples.

**Fig. 12:**
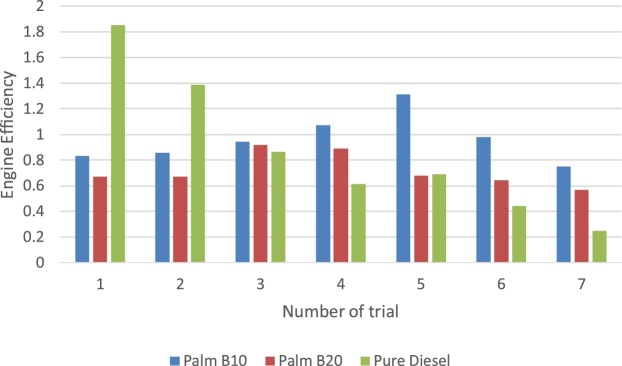
The engine efficiency of fuel samples.

**Fig. 13:**
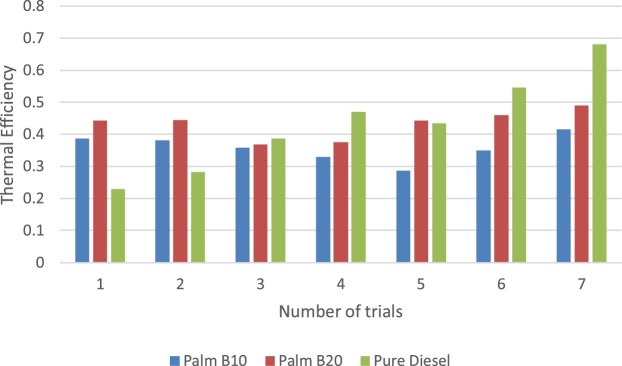
The thermal efficiency of fuel samples.

In this section, the derived parameters are presented to examine the experimental method beyond the primary dataset obtained from the TQ data display unit. The break mean effect pressure (BMEP) in engine is presented in Fig. 11. The pattern of the three samples considered is synonymous to the pattern of the power loss by the passive area of the engine. Though the biodiesel samples were lower, its difference with the pure petro-diesel was less than 10%. Muralidharan et al. [8] affirmed that the decreased engine brake power was as a result of uneven combustion of biodiesel product. The thermal and engine efficiency results are presented in Fig. 12, Fig. 13 respectively. It is observed that while the engine efficiency of pure diesel decreases with time of use, the biodiesel blend describes a positive parabolic pattern. This outcome gives the biodiesel advantage over the pure diesel. The pure petro-diesel sample produced the highest thermal efficiency and lowest engine efficiency. It is observed that this method was able to show that the thermal efficiency of the pure diesel increases with time of use while the biodiesel fluctuates due to cyclic breaking.

## Conflict of interest

The authors declare that there are no conflicts of interest.
